# *Streptococcus agalactiae* Serotype VII, an Emerging Pathogen Affecting Snakeskin Gourami (*Trichogaster pectoralis*) in Intensive Farming

**DOI:** 10.1155/2023/1682047

**Published:** 2023-03-24

**Authors:** Theeraporn Pulpipat, Visanu Boonyawiwat, Pattra Moonjit, Arsooth Sanguankiat, Sakuna Phatthanakunanan, Siriluk Jala, Win Surachetpong

**Affiliations:** ^1^Department of Farm Resources and Production Medicine, Faculty of Veterinary Medicine, Kasetsart University, Kamphaeng Saen Campus, Nakorn Pathom 73140, Thailand; ^2^Department of Veterinary Public Health, Faculty of Veterinary Medicine, Kasetsart University, Kamphaeng Saen Campus, Nakorn Pathom 73140, Thailand; ^3^Kamphaeng Saen Veterinary Diagnostic Center, Faculty of Veterinary Medicine, Kasetsart University, Kamphaeng Saen Campus, Nakorn Pathom 73140, Thailand; ^4^Department of Veterinary Microbiology and Immunology, Faculty of Veterinary Medicine, Kasetsart University, Bangkok 10900, Thailand

## Abstract

Snakeskin gourami (*Trichogaster pectoralis*) is a freshwater fish species that is being increasingly cultivated in Southeast Asia. The expansion of farms and intensive farming practices has led to the unexplained mortality of snakeskin gourami and tremendous economic losses in many farms. We investigated the unusual mortality of snakeskin gourami at 22 farms in Central Thailand. The moribund fish showed darkened skin, erratic swimming, exophthalmos, and haemorrhaging around the eyeballs, with cumulative mortality between 20% and 45%. Our necropsy findings revealed an enlarged liver and anterior kidney, splenomegaly, haemorrhage in most internal organs, pericarditis, and brain congestion. Histopathology revealed haemorrhaging and congestion of the blood vessels in the liver with infiltration of lymphocytes, enlarged blood vessels with mononuclear and lymphocyte infiltration in the meninges, and cerebral parenchyma were observed. Severe necrotising and suppurative pericarditis with myocardial infarction were found. Epidemiological studies and laboratory diagnosis revealed that *Streptococcus agalactiae* was predominantly isolated from the moribund fish. Laboratory investigations of the representative 33 isolates of *S. agalactiae* using mass spectrometry, multiplex polymerase chain reaction assay, pulse-gel electrophoresis, and serotyping suggested that all the isolates were *S. agalactiae* serotype VII, which is different from the serotype III isolated from diseased tilapia in Thailand. An experimental challenge using three representative isolates of *S. agalactiae* on snakeskin gourami caused clinical signs, gross lesions, and pathological changes, with high mortality exceeding 60%, which is similar to the mortality in most natural infections. Moreover, *S. agalactiae* was recovered from the spleen, kidneys, and liver of all the challenged fish. Taken together, this study provides important information that *S. agalactiae* serotype VII is virulent in snakeskin gourami and can potentially spread among these fish in fish farms. Appropriate preventive measures and the control of animal movements should thus be implemented.

## 1. Introduction

Snakeskin gourami (*Trichogaster pectoralis*) is one of the most important freshwater fish commonly cultured in Southeast Asia, including Laos, Vietnam, Cambodia, Malaysia, Indonesia, and Thailand [1–4]. Snakeskin gourami can tolerate a wide range of farming conditions as they have a special breathing organ, the labyrinth, which allows them to exchange oxygen in low oxygen cultivation conditions [5]. The production of snakeskin gourami has expanded dramatically over the last decade, and it has become one of the most important freshwater species in Thailand [6]. The growing demand for this fish and the expansion of intensive farming practices have led to sporadic outbreaks of infectious diseases, including bacterial infections [7–11]. Among these bacterial diseases, *Streptococcus* infection has been shown to cause high fish morbidity and extensive economic losses, which are affecting snakeskin gourami farmers [1].

Streptococcosis is caused by Gram-positive bacteria that cause diseases in many fish species. These bacteria include *S. agalactiae* (Group B *Streptococcus*, GBS), *S. iniae*, and *S. dysgalactiae* [11–14]. They can infect different species of fish, including marine, brackish, and freshwater fish, and lead to septicaemia and high mortality [11, 14, 15]. In general, the clinical signs and pathological lesions evident during infection by *Streptococcus* spp. are erratic swimming, unilateral or bilateral exophthalmos, cornea opacity, abdominal distension, hyperaemia, and skin haemorrhages [11, 16, 17]. As various fish species share water resources and are generally cultured in polyculture ponds, different *Streptococcus* spp. may spread from other fish species and cause disease in snakeskin gourami. Ten serotypes of *S. agalactiae*, namely, Ia, Ib and II–IX, have been characterised based on capsular antigens [18–22]. Three *S. agalactiae* serotypes (Ia, Ib, and III) are the main causes of *Streptococcus* infection in fish species in Thailand [19, 20, 23, 24]; however, *S. agalactiae* infection has not been reported in snakeskin gourami in Thailand or any other countries. A recent study reported *S. suis* infection in snakeskin gourami, which is a common *Streptococcus* spp. that infects swine and other species [1]. Notwithstanding, there have been no reports of other *Streptococcus* spp. in snakeskin gourami.

During the period July 2020‒May 2022, reports of abnormal mortality in pond-raised snakeskin gourami in provinces in Thailand. We, therefore, aimed to investigate the aetiology of this unusual mortality among snakeskin gourami at fish farms in Thailand. We isolated and characterised 33 isolates of *S. agalactiae* from snakeskin gourami at 22 farms in Thailand. Importantly, the virulence and pathogenicity of the *S. agalactiae* isolated from naturally infected snakehead gourami were confirmed by Koch's postulates under a laboratory challenge condition.

## 2. Materials and Methods

### 2.1. Fish Sampling and Bacteria Isolation

Samples of moribund snakeskin gourami were collected from 22 intensive fish farms located in Samut Sakhon, Samut Songkhram, and Phetchaburi provinces between July 2020 and May 2022 (Supplementary Figure S1). The fish were reared at a stocking density of 20,000–93,750 fish/acre in earthen ponds and fed with 28% protein commercial feed at 3% body weight daily. The cumulative mortality ranged from 20% to 45%, with body weight of 80–375 g. The water quality parameters were temperature 28°C–30°C, pH 7.5–8.3, dissolved oxygen 3–8 ppm, total ammonia 0.5–3.0 ppm, and nitrite 0.2–1.0 ppm.

A total of 10 fish were randomly collected from each outbreak of infection and submitted to the aquatic diagnostic unit at the Kamphaeng Saen Veterinary Diagnostic Center, Faculty of Veterinary Medicine, Kasetsart University, for clinical examination, necropsy, and histopathological and bacteriological examination. The bacteria were aseptically isolated from the liver, spleen, kidneys, and brain of each moribund fish, streaked on tryptic soy agar (TSA), and incubated at 28°C for 24–48 h to acquire the pure isolates. The details of the bacterial isolates, the collecting farms, and their geographic locations are presented in Supplementary Table S1. The bacteria were preserved in tryptic soy broth (TSB) with 20% glycerol (v/v) and stored at −80°C. The animal use protocol was approved by the institutional animal care and use committee under protocol number ACKU65-VET-011.

### 2.2. Histopathology

Tissues samples from the liver, anterior kidney, spleen, heart, and brain were collected from each moribund fish. Each 1 × 1 cm sample size was preserved in 10% buffered formalin for 24 h and then transferred to 70% ethanol for 48 h. The samples were embedded in a paraffin block, cut at 4 *μ*M thickness by a microtome (Thermo Scientific Shandon Finesse ME Microtome, USA), and stained with haematoxylin and eosin. The slides were examined under a light field microscope (Olympus CX43RF, Olympus, Japan), and photos were taken using a digital camera (MshOt MS60, Guangzhou Microshot Optical Technology Co., Ltd., China).

### 2.3. Bacterial Identification

The purified bacterial colony was subjected to Gram staining and bacterial identification using the automated mass spectrophotometry model Vitek® MS V3.2 (bioMérieux Marcy l'Etoile, France). Briefly, a single colony of bacteria, cultured on TSA, supplemented with 5% goat blood, was picked and mixed with 1 *μ*L of a Vitek® MS-CHCA matrix solution on a Vitek® MS target slide containing 48 spots. The samples plus the negative control were dried for 5 min and analysed using a Vitek® MS engine.

The species of bacteria was further confirmed by a multiplex polymerase chain reaction (PCR) assay using primer specific for *S. agalactiae* [25], *S. iniae* [26], and *Lactococcus garvieae* [27] by applying a protocol modified from [28]. The genomic DNA was extracted from an isolated bacterial colony using FavorPrep™ DNA Extraction Mini Kit (FAVORGEN Biotech Corporation, Taiwan). The PCR reaction consisted of 1 × DreamTaq Green PCR Master Mix (Thermo Scientific™, USA), 0.2 *μ*M of each primer pair for *S. agalactiae* and *S. iniae*, and 0.4 *μ*M for *L. garvieae*. The primers used in this study are listed in Supplementary Table S2. The PCR condition comprised a predenaturation step at 94°C for 4 min, followed by 35 cycles of denaturation at 94°C for 30 s, annealing at 58°C for 1 min, and an extension at 72°C for 2 min with a final extension of 72°C for 10 min. The amplification products were separated on 1.5% agarose gel, stained with RedSafe™ (iNtRON Biotechnology, Korea), and visualised under UV light. The positive controls used in this study were *S. agalactiae*, *S. iniae*, and *L. garvieae*, all of which had previously been characterised by the Kamphaeng Saen Veterinary Diagnostic Center. The negative control was DNase free water.

### 2.4. 16S rRNA Sequencing and Phylogenetic Tree Construction

Three representative isolates of *S. agalactiae* from the moribund snakeskin gourami from three geographical locations (Samut Sakhon, Samut Songkhram, and Phetchaburi) were selected and processed for 16S rRNA sequencing analysis. Briefly, the DNA was extracted from the bacteria and amplified using the universal primers EubA and EubB [29] (Supplementary Table S2) to generate multiple 1,500 bp fragments of 16S rRNA fragments. The PCR master mix comprised 1 × DreamTaq Green PCR Master Mix (Thermo Scientific™, USA) at 0.2 *μ*M for each primer. The PCR cycling conditions were predenaturation at 95°C for 3 min, followed by 35 cycles of denaturation at 95°C for 30 s, annealing at 52°C for 30 s, and an extension at 72°C for 1 min, with a final extension at 72°C for 10 min. The PCR products were separated on 1.5% agarose gel electrophoresis and stained with RedSafe™ (iNtRON Biotechnology, Korea). The PCR products were then purified using the FavorPrep™ GEL/PCR Purification Kit (FAVORGEN Biotech Corporation, Taiwan) and submitted for Sanger sequencing using 3500xL Genetic Analyzers (Applied Biosystems™, USA). The raw sequences were assembled using Contig Express software, subjected to BLAST in the NCBI nucleotide database, and aligned with those of the reference strains, namely, *S. agalactiae*, *S. iniae*, *S. dysgalactiae*, *S. suis*, and *L. garvieae* in the GenBank database (https://www.ncbi.nlm.nih.gov/nucleotide/) using the ClustalW program of the BioEdit software package. The phylogenetic tree was constructed using the neighbour-joining and 1,000 bootstrap replicates generated by MEGA-X software [30].

### 2.5. Molecular Serotyping and Analysis of Virulence Gene Profiling

#### 2.5.1. Molecular Capsular Serotyping

The DNA extracted from the bacterial samples isolated from the snakeskin gourami (*n* = 33) was subjected to a multiplex PCR assay using a primer set specific to the capsular polysaccharide (cps) genes of *S. agalactiae* [18] (Supplementary Table S2). The PCR condition was predenaturation at 95°C for 5 min, followed by 15 cycles of denaturation at 95°C for 1 min, annealing at 54°C for 1 min, an extension at 72°C for 1 min and, then by additional 25 cycles of denaturation at 95°C for 1 min, annealing at 56°C for 1 min, an extension at 72°C for 1 min, and a final extension at 72°C for 10 min. The amplification products were separated on 1.5% agarose gel, stained with RedSafe™ (iNtRON Biotechnology, Korea), and visualised under UV light.

#### 2.5.2. Virulence Gene Profiling

The pattern of the virulence genes in the *S. agalactiae* isolated from the snakeskin gourami was analysed using a multiplex PCR assay with primers specific to the five virulence genes (Supplementary Table S2), namely, *β*-haemolysin/cytolysin (cylE) [19], hyaluronate lyase (hylB) [19], C5a peptidase (scpB) [31], serine protease CspA (cspA) [19], and CAMP factor (cfb) [22]. The PCR condition included predenaturation at 95°C for 5 min, followed by 35 cycles of denaturation at 95°C for 30 s, an annealing temperature at 55°C for 30 s in each gene, an elongation at 72°C for 1 min, and a final elongation step at 72°C for 10 min. The amplified products were separated on 2% agarose gel, and specific bands were detected using RedSafe™ (iNtRON Biotechnology, Korea) staining and visualised under UV light.

### 2.6. Pulse-Field Gel Electrophoresis Pattern

A total of 22 representative isolates of *S. agalactiae* from 22 snakeskin gourami farms and one isolate of *S. agalactiae* from each of red tilapia and Nile tilapia were analysed by pulsed-field gel electrophoresis (PFGE) to distinguish the bacterial genotypes [32]. The PFGE results of the isolates were compared to assess the determined relatedness and genetic diversity, as shown in Table 1. The fresh bacterial colony was grown on a brain heart infusion agar (BHIA) and incubated at 28°C for 24 h. The purified bacterial colony was collected using a sterile cotton swab and resuspended in 1 mL phosphate buffer saline (PBS). The cell density was adjusted to McFarland No. 6, and 500 *μ*L of the bacterial suspension was transferred to a 1.5 mL tube and centrifuged at 5,000 rpm for 5 min to collect the bacterial pellet. The pellet was then washed three times with 500 *μ*L of PBS. Bacterial sample plugs were prepared using a 1 : 1 mixture of bacterial suspension and 2% (w/v) low-melting temperature agarose gel (SeaPlaque® GTG® Agarose, Cambrex Bio Science Rockland, Inc., USA). After solidification, the bacterial plugs were treated with a lysis buffer solution (5 mg/mL lysozyme, 0.5 M ethylenediaminetetraacetic acid (EDTA), 1% (w/v) sodium lauroyl sarcosine), incubated at 37°C for 24 h, and subsequently washed three times with reverse osmosis water. The bacterial plugs were then treated with an EDTA/Sarcosyl/Proteinase K solution (1 mg/mL proteinase K, 0.5 M EDTA, 1% (w/v) sodium lauroyl sarcosine) at 50°C for 24 h and washed twice with TE buffer (Tris-Cl, 0.5 M EDTA) for 1 h and stored in 70% ethanol plus the TE buffer at 4°C until use. PFGE was performed by digesting the bacterial plugs with the restriction enzyme SmaI (25 U) at 25°C for 5 h. The bacterial plugs were then transferred to 1% (w/v) agarose gel (Pulsed Field Certified Agarose, BioRad Laboratories, USA). PFGE was performed using a CHEF Mapper system (BioRad Laboratories) in 0.5 × TBE buffer under the following conditions: run time 22 h, switching time 10–45 s (linear ramping factor) at 14°C. The PFGE pulsotype patterns were analysed, and dendrograms were generated using GelCompar® II version 6.5 software (Applied Maths, Belgium).

### 2.7. Antimicrobial Susceptibility Profiling

The antimicrobial susceptibility of the *S. agalactiae* isolated from the snakeskin gourami was analysed using the Kirby‒Bauer disk diffusion method, as described in the Clinical and Laboratory Standards Institute (CLSI) [33]. The bacteria were cultured on TSA and incubated at 28°C for 24 h. The bacteria were harvested and suspended in a sterile 0.85% NaCl solution. The turbidity was measured as being equal to a McFarland No. 0.5 standard solution, and the bacteria were then spread on a Mueller Hinton agar plate with 5% sheep blood (Oxoid™, UK). The antimicrobial discs used in this study were amoxicillin (10 *μ*g), erythromycin (15 *μ*g), oxytetracycline (30 *μ*g), doxycycline (30 *μ*g), enrofloxacin (5 *μ*g), and sulfamethoxazole-trimethoprim (25 *μ*g). The antimicrobial discs (Oxoid, UK) were placed on a Mueller Hinton agar plate with 5% sheep blood using the Oxoid antimicrobial disc dispenser (Oxoid, UK) and incubated at 28°C for 24 h. After incubation, the diameter of the inhibition, which was designated as the clear zone, was measured and interpreted according to zone diameter breakpoints of *Streptococcus* spp. *β*-hemolysis group (amoxicillin, erythromycin, enrofloxacin, doxycycline, and oxytetracycline) and *Streptococcus pneumoniae* (sulfamethoxazole-trimethoprim) as defined in the CLSI guidelines [33]. Additionally, *Streptococcus pneumoniae* strain ATCC® 49619 was used as a reference strain, in accordance with the standard protocol of the CLSI guideline.

### 2.8. Experimental Challenge Study

We preliminarily assessed the median lethal dose (LD_50_) of all three representative isolates of *S. agalactiae* used in this study following the described protocol [34]. Briefly, each isolate was grown on TSA medium at 28°C for 24 hours, then collected, resuspended in sterile PBS, and adjusted to a concentration of 1 × 10^9^ CFU/mL using absorbance at OD600 = 1. The bacterial suspensions were then serially diluted 10-folds to reach concentrations of 10^8^, 10^7^, 10^6^, and 10^5^ CFU/mL. Each bacterial suspension was intracoelomically injected into 10 snakeskin gourami, and cumulative mortality was recorded for 21 days and LD_50_ concentration was calculated. For the challenge study, a total of 130 healthy snakeskin gourami with an average weight of 30 g were acquired from a farm with a *Streptococcus*-free history. Prior to the challenge study, necropsies were performed on 10 fish, which were screened for the presence of *Streptococcus* spp. via bacterial isolation on a TSA supplemented with 5% sheep blood and a multiplex PCR assay. The remaining 120 fish were divided into four groups and maintained in 180 L glass tanks with 30 fish per tank. The fish were allowed to acclimatise for two weeks at 28°C and fed with commercial feed at 3% body weight. Three isolates of *S. agalactiae*, namely, KU63SA1, KU63SA2, and KU64SA10, were used in the challenge study. The bacteria were cultured on TSA at 28°C for 24 h, collected, and resuspended in sterile PBS, before being adjusted to 1 × 10^9^ CFU/mL with the absorbance of 1 at OD_600._ The fish were anaesthetised using a Eugenol solution (Aquanes®, Betagro, Thailand) at 100 ppm for 3 min and then intraceolomic (i.c.)-injected with 0.1 mL of diluted bacterial suspension at 10^7^ CFU/mL, with the final concentration of bacteria injected at 10^6^ CFU/fish. The control fish (*n* = 30) were i.c.-injected with 0.1 mL of sterile PBS. The cumulative mortality, clinical signs, and gross lesions were monitored and recorded for 21 days after the injection. At day 3 postchallenge, three moribund fish were randomly collected from each group for necropsy and bacterial isolation. Tissue samples (spleen, kidney, liver, brain, and heart) were collected from the moribund fish and processed for histopathology. The bacterial colonies isolated from the experimentally challenged fish were confirmed via a species-specific multiplex PCR assay, as described in Section 2.3.

## 3. Results

### 3.1. Disease Investigation and Pathology of Moribund Snakeskin Gourami

**Figure 1:**
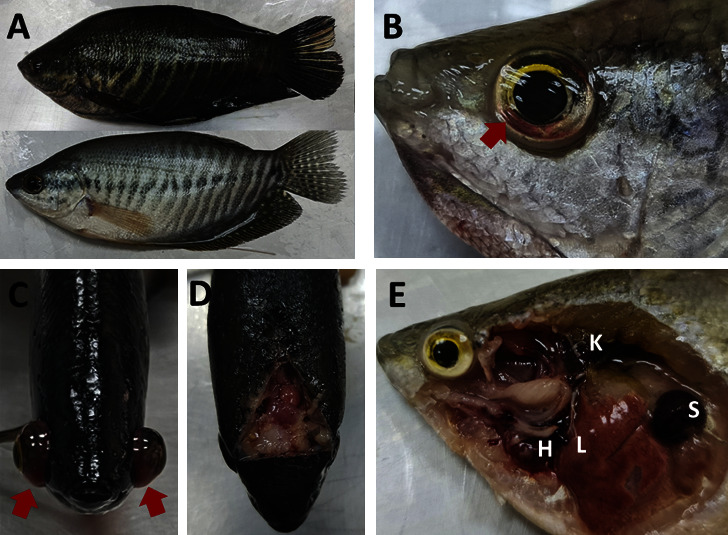
External appearance and gross lesions caused by natural *Streptococcus agalactiae* infection in snakeskin gourami: (a) darkening skin, (b) eye congestion (red arrow), (c) bilateral exophthalmia (red arrow), (d) brain congestion (white arrow), and (e) haemorrhagic liver (L), enlarged anterior kidney (K), splenomegaly (S), and white-greyish and purulent mass in the heart (pericarditis) (H).

**Figure 2:**
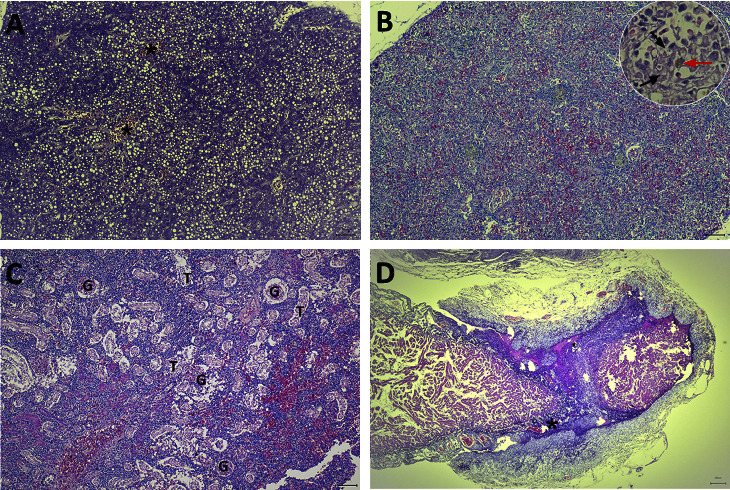
Histopathological findings of natural *Streptococcus agalactiae* infection in snakeskin gourami. (a) Liver: congestion (asterisk), area of haemorrhage, and vacuolation of hepatocytes. (b) Spleen: bacteria trapped in ellipsoids (black arrow) and brown pigment derived from lysed melanomacrophages (red arrow). (c) Anterior kidney: acute glomerulonephritis with bacteria, particles in glomeruli (G), degeneration, and necrosis of the tubular epithelium with inflammatory cell infiltration in the glomerulus (T). (d) Heart: pericarditis with lymphocyte infiltration and necrotising suppurative pericarditis and endocarditis with myocardial infarction (black asterisk).

From July 2020 to May 2022, a total of 22 snakeskin gourami farms, distributed across three provinces in Thailand, were investigated to determine the cause of unusual fish mortality (Supplementary Table S1). A total of 220 fish (10 fish/farm) were processed to investigate the disease. The most common clinical signs of the diseased fish were feeding cessation, swimming at the water surface, erratic swimming, and daily mortality above 3%–10% continuously for 3–5 days. The gross findings of the moribund fish included darkening skin (Figure 1(a)), congestion around the eyes (Figure 1(b)), and unilateral or bilateral exophthalmos (Figure 1(c)). The necropsy findings showed brain congestion (Figure 1(d)), empty intestines, spleen and kidney enlargement and moderate-to-severe liver congestion, and white-greyish and purulent-mass in the heart (Figure 1(e)). Histologically, the livers of the moribund fish showed severe congestion and lymphocyte infiltration (Figure 2(a)). In the spleen, bacteria trapped in ellipsoids and an increased melanomacrophage centre area with brown pigments of lysed bacteria and cells were commonly observed (Figure 2(b)). Acute glomerulonephritis, as demonstrated by the infiltration of inflammatory cells and thickening of the glomeruli, together with degeneration and necrosis of the tubular epithelium and congestion, were noticed in most of the moribund fish (Figure 2(c)). In the heart, the main pathological changes included severe necrotising and suppurative pericarditis with myocardial destruction (Figure 2(d)). The clinical parasite examinations revealed that some of the moribund fish were infested with ectoparasites: *Trichodina* spp. (9/22 farms), *Gyrodactylus* spp. (1/22 farms), and *Henneguya* spp. (3/22 farms). No endoparasites were found in any of the necropsied fish.

### 3.2. Bacterial Identification

**Figure 3:**
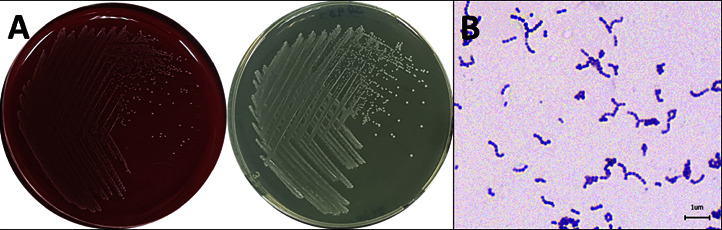
Morphology and Gram's stain of *Streptococcus agalactiae* isolated from snakeskin gourami. (a) Colonies of *S. agalactiae* on a tryptic soy agar (TSA) supplemented with 5% of sheep blood agar (left) and TSA agar (right) showing small white mucoid pinpoint colonies. (b) Gram-positive cocci.

In our study, a comprehensive examination of 220 diseased fish collected from 33 ponds across 22 farms, with 10 fish sampled from each farm was conducted to determine the cause of mortality, the bacteria were isolated from the livers, spleens, kidneys, and brains of the moribund fish. Bacterial colonies with creamy, round morphology were isolated from moribund snakeskin gourami using a TSA medium supplemented with 5% sheep blood and TSA. The majority of the isolated colonies exhibited white, mucoid appearance with small, pinhead-sized dimensions (Figure 3). Following characterization, 152 isolates of small, white colonies were identified as Gram-positive cocci (Figure 3), exhibiting *β*-hemolysis and catalase negativity, which led to the tentative classification of the isolates into the genus *Streptococcus.* To identify the species, a representative 33 isolates from different 33 ponds were selected to identify the species using multiplex PCR with specific primers against *S. agalactiae*, *S. iniae*, and *L. garvieae.* The presence of a specific 220 bp band on gel electrophoresis confirmed the identification of these isolates as *S. agalactiae* (Supplementary Figure S2). Based on mass spectrophotometry analysis, these bacterial samples from the moribund snakeskin gourami were confirmed as *S. agalactiae* with 99.9% confidence. Besides *S. agalactiae*, other bacteria, including *Aeromonas* spp. and *Flavobacterium columnare*, were isolated from some of the samples collected in this study (Supplementary Table S1).

### 3.3. 16S rRNA Gene Sequencing and Phylogenetic Tree

**Figure 4:**
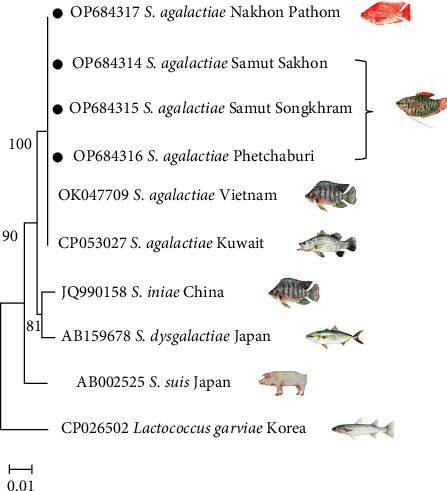
Neighbour-joining trees generated from a nearly full-length 16S rRNA gene sequence (1,362 bp) of *S. agalactiae* isolate from snakeskin gourami (accession number OP684314–OP684316), another fish strain of *S. agalactiae* from red tilapia in Thailand (accession number OP648317), tilapia from Vietnam (accession number OK047709), seabass from Kuwait (accession number CP053027), and their closely related species *S. iniae* (accession number JQ990158), *S. dysgalactiae* (accession number JQ990158), *S. suis* (accession number AB002525), and *Lactococcus garvieae* (accession number CP026502). The number next to the branch represents the percentage bootstrap value (1,000 replicates). The black dots highlight the bacteria in this study.

The partial 16S rRNA gene sequences of the representative *S*. *agalactiae* strains KU63SA1 (accession number OP648314), KU63SA2 (accession number OP648315), and KU64SA10 (accession number OP648316) were deposited and compared with existing sequences in the GenBank database. BLAST analysis revealed that all three representative isolates had a 99.93%–100% match to other *S. agalactiae* from tilapia and seabass, but none of the *S. agalactiae* that appeared in the GenBank database had been isolated from snakeskin gourami. The construction of a phylogenetic tree based on the 16S rRNA gene *S. agalactiae* showed that all the *S. agalactiae* isolates from the snakeskin gourami in this study were clustered with the *S. agalactiae* previously isolated from red tilapia (*Oreochromis* spp.) in Thailand (accession number OP684317), tilapia (*Oreochromis* spp.) from Vietnam, and seabass (*Lates calcarifer*) from Kuwait (Figure 4). A comparison showed that the *S. agalactiae* isolated from the snakeskin gourami was different and separate from the *Streptococcus* reported in other species, including *S. iniae* from tilapia (*Oreochromis* spp.), *S. dysgalactiae* from yellowtail amberjack (*Seriola lalandi*), *S. suis* from swine (*Sus domesticus*), and *L. garvieae* from redlip mullet (*Liza haematocheila*).

### 3.4. Molecular Serotyping, Virulence Gene Profiling, and PFGE Analysis

**Figure 5:**
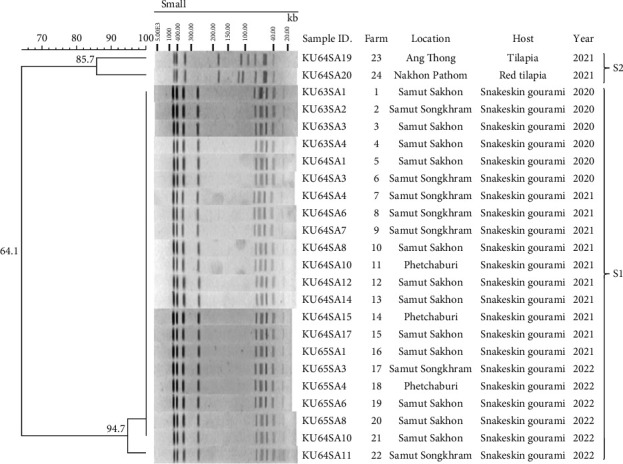
Dendrogram of *S. agalactiae* isolated from snakeskin gourami and tilapia cut by the SmaI restriction enzyme. Twenty-two isolates of *S. agalactiae* from the diseased snakeskin gourami in this study and two isolates of *S. agalactiae* from tilapia were stored in our pathogen banks. Bacterial genomes were cut by the *Sma*I restriction enzyme, separated by pulse field gel electrophoresis, and analysed by unweighted pair group method with arithmetic mean (UPGMA) cluster analysis using GelCompar® II software.

Molecular serotyping of the representative *S. agalactiae* (*n* = 33) isolated from the moribund snakeskin gourami revealed that all the isolates were serotype VII, while the *S. agalactiae* previously isolated from diseased Nile tilapia and red hybrid tilapia in Thailand were serotype III (Table 1, Supplementary Figure S3). Interestingly, *S. agalactiae* serotype VII, which causes disease in snakeskin gourami, and serotype III from tilapia showed similar virulence gene profiles as they both contained *cyl*E, *hyl*B, *scp*B, *csp*A, and *cfb* (Table 1). All of these virulence genes have been previously shown to be responsible for the pathogenicity and tissue damage. The PFGE cluster analysis revealed that the representative 22 isolates of *S. agalactiae* obtained from 22 different farms of diseased snakeskin gourami showed a similar pattern of chromosomal DNA after digestion by *Sma*I, with nine fragments sized between 23 and 550 kb (Figure 5). In contrast, *S. agalactiae* serotype III isolated from tilapia and red tilapia showed a pattern of nine fragments of 40–500 kb. An analysis of the PFGE pattern from all 22 isolates of *S. agalactiae* from the snakeskin gourami showed 94.7% similarity and was designated as an S1 pulsotype, while the PFGE pattern of *S. agalactiae* from tilapia had 85.7% similarity and was designated as an S2 pulsotype (Figure 5).

### 3.5. Antimicrobial Susceptibility Test

All 33 isolates of *S. agalactiae* from the snakeskin gourami were subjected to antimicrobial susceptibility testing with amoxicillin, erythromycin, enrofloxacin, oxytetracycline, doxycycline, and sulfamethoxazole-trimethoprim. As shown in Table 2, all the *S. agalactiae* were susceptible to amoxycillin and erythromycin. The majority (96.69%) of the *S. agalactiae* from the snakeskin gourami, with one resistant isolate, were susceptible to enrofloxacin. The result of the antimicrobial susceptibility test to tetracycline drugs (oxytetracycline and doxycycline) was moderate to high. A total of 30 isolates were susceptible to doxycycline, two isolates (6.06%) were moderately resistant, and another isolate (3.03%) was resistant to doxycycline. Notably, 27 isolates (81.82%) of *S. agalactiae* were susceptible to oxytetracycline, although six isolates (18.18%) were moderately resistant to oxytetracycline. Notably, only 15 isolates (45.54%) of *S. agalactiae* showed susceptibility to sulfamethoxazole trimethoprim. The antimicrobial susceptibility results of a reference *S. pneumoniae* strain ATCC® 49619 are provided in Supplementary Table S3.

### 3.6. Challenge Study

**Figure 6:**
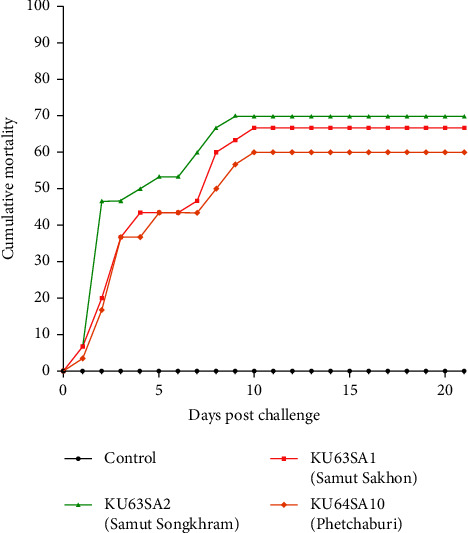
Cumulative mortality of snakeskin gourami experimentally challenged by three isolates of *S. agalactiae* SA63SA1 (Samut Sakhon), SA63SA2 (Samut Songkhram), and SA64SA10 (Phetchaburi).

**Figure 7:**
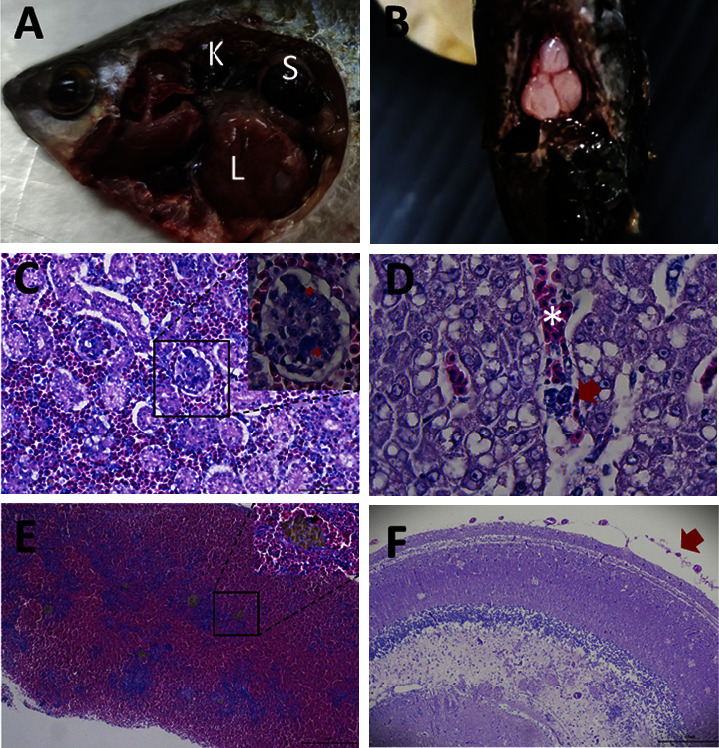
Gross lesions and histopathology of the fish experimentally challenged by *S. agalactiae* at 3 days postchallenge. (a) Necropsy findings of the internal organs of the challenged fish: splenomegaly (S), enlarged anterior kidney (K), liver congestion (L). (b) Brain congestion. (c) Anterior kidney: congestion of the blood vessel surrounding the glomeruli, glomerulonephritis with clump of cocci bacteria in the glomeruli (red arrow). (d) Liver: congestion of the sinusoid (white asterisk) with bacteria clump (red arrow). (e) Spleen: congestion of the blood vessels and accumulation of the melanomacrophage centres. (f) Brain: thin and blood congestion in the meninges (red arrow), infiltration of inflammatory cells (lymphocytes and macrophages) in the brain tissue.

Intracoelomic injections of three representative isolates of *S. agalactiae*, namely, KU63SA1 (Samut Sakhon), KU63SA2 (Samut Songkhram), and KU64SA10 (Phetchaburi), were administered to healthy snakeskin gourami at a final concentration of 8.4 × 10^6^ CFU/fish, 8.35 × 10^6^ CFU/fish, and 8.02 × 10^6^ CFU/fish, respectively, in accordance with the respective LD_50_ dose of each isolate (Supplementary Table S4). The LD_50_ of all three representative strains were evaluated and ranged between shown in Supplementary Table S4. At two dpc, the moribund fish developed clinical signs of lethargy and loss of appetite, while some fish showed erratic swimming and swimming near the water surface at 5 dpc. Mortality started at 1 dpc and continued until 21 dpc, with the cumulative mortality at 60%, 70%, and 66.67% in the fish inoculated with KU63SA1, KU63SA2, and KU64SA10, respectively (Figure 6). No mortality was observed in the control fish that received PBS injections. Gross findings that included a haemorrhagic liver (Figure 7(a)), an accumulation of blood congestion in the brain (Figure 7(b)), splenomegaly (Figure 7(a)), and an enlarged anterior kidney (Figure 7(a)) were consistently found in the fish inoculated with *S. agalactiae* KU63SA1, KU63SA2, and KU64SA1. Similar to natural infection, histopathological changes were found in multiple organs of the challenged fish and were consistent in the fish that received all three isolates of *S. agalactiae*. In the anterior kidney, severe congestion of the blood vessels surrounding the glomeruli was observed, as was glomerulonephritis with clumps of cocci bacteria (Figure 7(c)). Similarly, congestion of sinusoid and bacterial aggregation was widely distributed in the liver (Figure 7(d)). The common histopathological changes in other organs included increased melanomacrophage centres in the spleen (Figure 7(e)), severe congestion of the blood vessels in the meninges (Figure 7(f)), and the infiltration of inflammatory cells in the brain tissue (Figure 7(f)).

## 4. Discussion

*S. agalactiae* is an important bacterial disease that commonly causes high mortality in many fish species and massive economic losses among affected fish farmers [12]. In addition to tilapia, various fish species are susceptible to infection by *S. agalactiae* [12, 35–38]. To the best of our knowledge, no report or direct evidence exists of the virulence and pathogenicity of *S. agalactiae* in snakeskin gourami. A recent study showed that *S. suis* can lead to high mortality and death in naturally infected and experimentally challenged snakeskin gourami [1]. Our results provide the first comprehensive epidemiological study that *S. agalactiae* is pathogenic and causes high mortality in farmed snakeskin gourami. They further highlight the importance of this bacteria in another susceptible fish species. Our gross findings and the histopathological changes in naturally infected snakeskin gourami showed pathological changes that were similar to *S. agalactiae* infections in other fish species [12, 13, 24, 35–39]. The virulence of *S. agalactiae* isolated from snakeskin gourami was additionally confirmed in a laboratory challenge condition. The three selected bacterial isolates (KU63SA1, KU63SA2, and KU64SA10), which were deemed representative based on similarities in biochemical, molecular, and genetic characteristics and their correlation with outbreaks in specific provinces, resulted in similar clinical signs, mortality, and pathological findings in challenged fish compared to natural infection. Specifically, high mortality exceeding 60% and severe pathological changes in most internal organs were found in all the challenged groups, which confirmed the virulence and Koch's postulates of the bacteria. Moreover, the challenge bacteria could be recovered from the internal organs of the challenged fish at 3 dpc and verified as *S. agalactiae* using bacterial isolation and multiplex PCR analysis.

Further serotyping of the 33 isolates of *S. agalactiae* from moribund snakeskin gourami by multiplex PCR assay revealed that all the isolates belonged to the serotype VII. Notably, previous studies have shown that the five serotypes of *S. agalactiae* (Ia, Ib, III, IV, and IX) are pathogenic and cause high mortality in other fish species [15, 22, 24, 32, 36, 38–40]. Interestingly, although *S. agalactiae* serotype VII has not been reported to cause disease in fish, previous reports have described this serotype in humans in Southern Ghana, West Africa, Malaysia, Iran, and Bangladesh [41–45]. Additional characterisation of the bacteria using PFGE analysis confirmed *S. agalactiae* serotype VII in the moribund snakeskin gourami, which is distinct from the *S. agalactiae* serotype III isolated from tilapia and red tilapia. The virulence gene profiling, namely, *cly*E, *hyl*B, *scp*B, *cps*A, and *cfb*, was detected in all the isolates of *S. agalactiae* from the snakeskin gourami, which further confirmed that most of the bacteria isolated from the moribund fish in this epidemiological study were virulent and homogenous. Among these genes, *cyl*E and *cfb* contribute to tissue damage and facilitate bacterial entry into fish cells, while *hyl*B lyses red blood cells, *scp*B helps the bacteria evade the host immune system, and *csp*A cleaves host proteins to avoid detection by the immune system [46–49]. The identification of these virulence factors not only enhance our understanding of the pathogenesis of *S. agalactiae* but also holds promise for developing novel therapeutic strategies to combat infections caused by this bacteria [50, 51]. Previously, a similar pattern of these virulence genes was identified in the *S. agalactiae* that causes severe infection in Nile tilapia and red tilapia [19, 20], climbing perch (*Anabas testudineus*), and Günther's walking catfish (*Clarias macrocephalus*) [38].

To reduce the negative impact of *S. agalactiae* infection on intensive snakeskin gourami farming, many of the farmers who participated in our study added antimicrobial agents to the fish feed as treatment. However, most fish stop feeding during an outbreak, thus making the antimicrobial agents ineffective. In this study, six commonly used antimicrobial agents, namely amoxicillin, erythromycin, oxytetracycline, doxycycline, enrofloxacin, and sulfamethoxazole-trimethoprim were selected for the antimicrobial susceptibility test, as they are frequently used for the prevention and treatment of bacterial diseases in farmed fish in Thailand and other Asian countries [52, 53]. Of these, amoxicillin, oxytetracycline, enrofloxacin, and sulfamethoxazole-trimethoprim are approved for use in Thailand, while doxycycline is approved for use in China and Bangladesh, and erythromycin is approved for use in Japan, Bangladesh, and Chile [54]. Interestingly, antimicrobial susceptibility testing revealed that some isolates of *S. agalactiae* in this study were resistant to some antimicrobial agents, including sulfamethoxazole-trimethoprim, doxycycline, and enrofloxacin, which is used as a bacterial treatment in snakeskin gourami farms. Notwithstanding, improper antibiotic usage may lead to antibiotic-resistant bacteria, which is an important problem that could affect human health [55–59]. Importantly, further active epidemiological surveillance and proper control measures should be implemented to limit the impact of *Streptococcus* infection in snakeskin gourami.

In this study, several bacteria (*Aeromonas* spp. and *Flavobacterium columnare*) and ectoparasites (*Trichodina* spp., *Gyrodactylus* spp., and *Henneguya* spp.) were identified in moribund fish. However, based on the presence of clinical signs, gross lesions, and the isolation of *S. agalactiae* from the moribund snakeskin gourami in all 33 ponds of 22 farms, we believe that infection by *S. agalactiae* may have played a significant role in the mass mortality of farmed snakeskin gourami. It is important to note that other factors, such as poor water quality, inappropriate farm management, and other pathogens such as bacteria, parasites, and viruses, may have also contributed to the loss of fish. In tilapia, high temperatures could promote the virulence of *S. agalactiae* and lead to higher fish mortality [60–63]. In this study, most of the outbreaks of *S. agalactiae* were found during the summer and rainy season (March to August) when the average water temperature in Thailand ranges between 28°C and 33°C, and high temperature fluctuations occur during the day. Nonetheless, the contribution of temperature to the virulence of *S. agalactiae* in this fish species remains to be determined. Therefore, further investigation is necessary to fully understand the causes of mass mortality in farmed snakeskin gourami. A comprehensive disease investigation and pathogen characterization can provide valuable insights to identify the cause of fish mortality and guide appropriate control measures and vaccine development. For example, previous research has reported that PFGE and serotype screening are effective tools for developing vaccines against the serotype and pulsotype of *S. agalactiae* in tilapia [19, 64]. This approach can guide to the development of a *S. agalactiae*serotype-specific vaccine for snakeskin gourami. However, additional research and testing are necessary to comprehensively evaluate the efficacy of the vaccine and explore its potential for broader application beyond the outbreak. Developing a specific pathogen-causing vaccine depends on a range of factors, and therefore, further research and testing are necessary.

## 5. Conclusion

This is the first intensive investigation of *S. agalactiae* in farmed snakeskin gourami over a two-year interval in Thailand. The gross findings, pathological changes, bacterial characterisation, and laboratory challenge study revealed that *S. agalactiae* serotype VII caused high mortality and virulence in the snakeskin gourami in this study. Our findings provide important epidemiological information and pathology of *S. agalactiae* infection in snakeskin gourami and lay a solid foundation for further research on bacterial distribution and interactions with these fish.

## Figures and Tables

**Table 1:** Details of samples and characterization of *S. agalactiae.*

Location	Number of isolates	Host	Serotype	PFGE pulsotype	*Virulence genes profile*				
					*cly*E	*hyl*B	*scp*B	*cps*A	*cfb*
Samut Sakhon	19	Snakeskin gourami	VII	S1	+	+	+	+	+
Samut Songkhram	8	Snakeskin gourami	VII	S1	+	+	+	+	+
Phetchaburi	6	Snakeskin gourami	VII	S1	+	+	+	+	+
Ang Thong	1	Tilapia	III	S2	+	+	+	+	+
Nakon Pathom	1	Red tilapia	III	S2	+	+	+	+	+

**Table 2:** Summary of the antimicrobial susceptibility profiles of pathogenic *S. agalactiae* isolated from snakeskin gourami.

Antimicrobial agents	Susceptible (%)	Intermediate (%)	Resistance (%)
Amoxycillin (10 *μ*g)	100 (33/33)	0 (0/33)	0 (0/33)
Erythromycin (15 *μ*g)	100 (33/33)	0 (0/33)	0 (0/33)
Oxytetracycline (30 *μ*g)	81.82 (27/33)	18.18 (6/33)	0 (0/33)
Doxycycline (30 *μ*g)	90.90 (30/33)	6.06 (2/33)	3.03 (1/33)
Enrofloxacin (5 *μ*g)	96.69 (32/33)	0 (0/33)	3.03 (1/33)
Sulfamethoxazole-trimethoprim (25 *μ*g)	45.45 (15/33)	0 (0/33)	54.54 (18/33)

## Data Availability

The data used to support the findings of this study are available from the corresponding author upon request.

## Supplementary Materials

Supplementary Materials The supplementary file contains three figures and four tables. Figure S1. A map of Thailand showing the locations of sample collection. Figure S2. Identification of bacterial samples using a multiplex PCR assay with specific primers against *S. agalactiae*, *S. iniae*, and *Lactococcus garvieae*. Figure S3. Molecular serotyping of *S. agalactiae* using a multiplex PCR assay with specific primers based on capsular polysaccharide (cps) cluster gene. Table S1. Detail of locations, farming history, mortality, and other pathogens detected that were observed in moribund snakeskin gourami in this study. Table S2. List of primers used in this study. Table S3. Diameter size and interpretation of antimicrobial susceptibility test (zone of diameter breakpoint) and the zone diameter of the standard S. *pneumoniae* strain ATCC® 49619. Table S4. The median lethal dose (LD50) dose for three representative isolates of *S. agalactiae* (KU63SA1, KU63SA2, and KU64SA10).

## References

[1] Dinh-Hung N., Dong H. T., Taengphu S. (2023). Streptococcus suis is a lethal pathogen in snakeskin gourami. *Trichopodus pectoralis Aquaculture*.

[2] Klinmalai P., Fong S., Phongthai S., Klunklin W. (2021). Improving the quality of frozen fillets of semi-dried gourami fish (Trichogaster pectoralis) by using sorbitol and citric acid. *Foods*.

[3] Phuoc Minh N., Xuan Mai P., Van Linh N. T. (2019). Physical aspects influencing to the production of dry-salted snakeskin gourami (Trichogaster pectoralis). *Oriental Journal of Chemistry*.

[4] Ramadhani A. A. N., Kholilullah Z. A., Arifah S., Purwanto E., Omar S. B. A. (2020). Detection of lead (Pb) heavy metal in snakeskin gourami (*Trichopodus pectoralis*) meat in Lake Tempe, South Sulawesi. *IOP Conference Series: Earth and Environmental Science*.

[5] Phong N. (2014). Snakeskin gourami in the Mekong. *Aqua Culture Asia Pacific*.

[6] Fisheries statistics of Thailand (2020). Fisheries development policy and planning division.

[7] Pulkkinen K., Suomalainen L. R., Read A. F., Ebert D., Rintamäki P., Valtonen E. T. (2010). Intensive fish farming and the evolution of pathogen virulence: the case of columnaris disease in Finland. *Proceedings. Biological sciences*.

[8] Nhinh D. T., Le D. V., Van K. V., Huong Giang N. T., Dang L. T., Hoai T. D. (2021). Prevalence, virulence gene distribution and alarming the multidrug resistance of Aeromonas hydrophila associated with disease outbreaks in freshwater aquaculture. *Antibiotics*.

[9] Mzula A., Wambura P. N., Mdegela R. H., Shirima G. M. (2021). Present status of aquaculture and the challenge of bacterial diseases in freshwater farmed fish in Tanzania; A call for sustainable strategies. *Aquaculture and Fisheries*.

[10] Pridgeon J. W., Klesius P. H. (2012). Major bacterial diseases in aquaculture and their vaccine development. *CABI Reviews*.

[11] Piamsomboon P., Thanasaksiri K., Murakami A. (2020). Streptococcosis in freshwater farmed seabass Lates calcarifer and its virulence in Nile tilapia Oreochromis niloticus. *Aquaculture*.

[12] El-Noby A., Hassanin M., El-Hady M., Aboshabana S. (2021). Streptococcus: a review article on an emerging pathogen of farmed fishes. *Egyptian Journal of Aquatic Biology and Fisheries*.

[13] Amal M., Zamri-Saad M. (2011). Streptococcosis in tilapia (*Oreochromis niloticus*): a review. *Journal of Tropical Agricultural Science*.

[14] Van Doan H., Soltani M., Leitão A. (2022). Streptococcosis a Re-emerging disease in aquaculture: significance and phytotherapy. *Animals*.

[15] Chu C., Huang P.-Y., Chen H.-M. (2016). Genetic and pathogenic difference between *Streptococcus agalactiae* serotype Ia fish and human isolates. *BMC Microbiology*.

[16] Klesius P., Evans J., Shoemaker C. (2006). Rapid detection and identification of Streptococcus iniae using a monoclonal antibody-based indirect fluorescent antibody technique. *Aquaculture*.

[17] Anshary H., Kurniawan R. A., Sriwulan S., Ramli R., Baxa D. V. (2014). Isolation and molecular identification of the *etiological agents of streptococcosis in Nile tilapia (Oreochromis niloticus) cultured in net cages in Lake Sentani, Papua, Indonesia*. *Springerplus*.

[18] Imperi M., Pataracchia M., Alfarone G., Baldassarri L., Orefici G., Creti R. (2010). A multiplex PCR assay for the direct identification of the capsular type (Ia to IX) of 565 Streptococcus agalactiae. *Journal of Microbiological Methods*.

[19] Kannika K., Pisuttharachai D., Srisapoome P. (2017). Molecular serotyping, virulence gene profiling and pathogenicity of Streptococcus agalactiae isolated from tilapia farms in Thailand by multiplex PCR. *Journal of Applied Microbiology*.

[20] Kayansamruaj P., Pirarat N., Katagiri T., Hirono I., Rodkhum C. (2014). Molecular characterization and virulence gene profiling of pathogenic *Streptococcus agalactiae* populations from tilapia (*Oreochromis* sp.) farms in Thailand. *Journal of Veterinary Diagnostic Investigation*.

[21] Poyart C., Tazi A., Réglier-Poupet H. (2007). Multiplex PCR assay for rapid and accurate capsular typing of group B streptococci. *Journal of Clinical Microbiology*.

[22] Zhang Z., Lan J., Li Y. (2018). The pathogenic and antimicrobial characteristics of an emerging Streptococcus agalactiae serotype IX in Tilapia. *Microbial Pathogenesis*.

[23] Dangwetngam M., Suanyuk N., Kong F., Phromkunthong W. (2016). Serotype distribution and antimicrobial susceptibilities of *Streptococcus agalactiae* isolated from infected cultured tilapia (*Oreochromis niloticus*) in Thailand: nine-year perspective. *Journal of Medical Microbiology*.

[24] Suanyuk N., Kong F., Ko D., Gilbert G. L., Supamattaya K. (2008). Occurrence of rare genotypes of Streptococcus agalactiae in cultured red tilapia Oreochromis sp. and Nile tilapia O. niloticus in Thailand—relationship to human isolates?. *Aquaculture*.

[25] Martinez G., Harel J., Gottschalk M. (2001). Specific detection by PCR of Streptococcus agalactiae in milk. *Canadian Journal of Veterinary Research*.

[26] Mata A. I., Blanco M. M., Domínguez L., Fernández-Garayzábal J. F., Gibello A. (2004). Development of a PCR assay for Streptococcus iniae based on the lactate oxidase (lctO) gene with potential diagnosticvalue. *Veterinary Microbiology*.

[27] Zlotkin A., Eldar A., Ghittino C., Bercovier H. (1998). Identification of Lactococcus garvieae by PCR. *Journal of Clinical Microbiology*.

[28] Itsaro A., Suanyuk N., Tantikitti C. (2012). Multiplex PCR for simultaneous detection of Streptococcus agalactiae, Streptococcus iniae and Lactococcus garvieae: a case of S. agalactiae infection in cultured Nile tilapia (Oreochromis niloticus) and red tilapia (Oreochromis niloticus x Oreochromis mossambicus). *Journal of Science and Technology*.

[29] Kirchman D. L., Yu L., Cottrell M. T. (2003). Diversity and abundance of uncultured cytophaga-like bacteria in the Delaware estuary. *Applied and Environmental Microbiology*.

[30] Kumar S., Stecher G., Li M., Knyaz C., Tamura K. (2018). Mega X: molecular evolutionary genetics analysis across computing platforms. *Molecular Biology and Evolution*.

[31] Dmitriev A., Suvorov A., Shen A., Yang Y. (2004). Clinical diagnosis of group B streptococci by scpB gene based PCR. *Indian Journal of Medical Research*.

[32] Sudpraseart C., Wang P. C., Chen S. C. (2020). Phenotype, genotype and pathogenicity of *Streptococcus agalactiae* isolated from cultured tilapia (*Oreochromis* spp.) in Taiwan. *Journal of Fish Diseases*.

[33] Clinical and Laboratory Standards Institue (2020). CLSI supplement M100. *Performance Standards For Antimicrobial Susceptibility Testing*.

[34] Wilbrandt W. (1952). Behrens methods for calculation of LD50. *Arzneimittelforschung*.

[35] Abuseliana A. F., Mohd Daud H. H., Abdul Aziz S., Bejo S. K., Alsaid M. (2011). Pathogenicity of Streptococcus agalactiae isolated from a fish farm in Selangor to juvenile red tilapia (Oreochromis sp.). *Journal of Animal and Veterinary Advances*.

[36] Bowater R. O., Forbes-Faulkner J., Anderson I. G. (2012). Natural outbreak of streptococcus agalactiae (gbs) infection in wild giant queensland grouper, epinephelus lanceolatus (bloch), and other wild fish in Northern Queensland, Australia. *Journal of Fish Diseases*.

[37] Geng Y., Wang K. Y., Huang X. L. (2012). Streptococcus agalactiae, an emerging pathogen for cultured ya-fish, Schizothorax prenanti, in China. *Transboundary and Emerging Diseases*.

[38] Suanyuk C. K. N. (2017). *Streptococcus agalactiae* serotype Ib, an emerging pathogen affecting climbing perch (*Anabas testudineus*) and Günther’s walking catfish (*Clarias macrocephalus*) polycultured in southern Thailand. *Thai Journal of Veterinary Medicine*.

[39] Delannoy C. M. J., Samai H., Labrie L. (2021). Streptococcus agalactiae serotype IV in farmed tilapia. *Aquaculture*.

[40] Zhang D., Li A., Guo Y., Zhang Q., Chen X., Gong X. (2013). Molecular characterization of Streptococcus agalactiae in diseased farmed tilapia in China. *Aquaculture*.

[41] Slotved H.-C., Dayie N., Banini J., Frimodt-Møller N. (2017). Carriage and serotype distribution of *Streptococcus agalactiae* in third trimester pregnancy in southern. *BMC Pregnancy and Childbirth*.

[42] Eskandarian N., Ismail Z., Neela V., van Belkum A., Desa M. N. M., Amin Nordin S. (2015). Antimicrobial susceptibility profiles, serotype distribution and virulence determinants among invasive, non-invasive and colonizing Streptococcus agalactiae (group B streptococcus) from Malaysian patients. *European Journal of Clinical Microbiology and Infectious Diseases*.

[43] Islam M. S., Saha S. K., Islam M. (2016). Prevalence, serotype distribution and mortality risk associated with group B *Streptococcus* colonization of newborns in rural Bangladesh. *Journal of Pediatric Infectious Diseases*.

[44] Motallebirad T., Fazeli H., Ghahiri A. (2021). Prevalence, population structure, distribution of serotypes, pilus islands and resistance genes among erythromycin-resistant colonizing and invasive Streptococcus agalactiae isolates recovered from pregnant and non-pregnant women in Isfahan, Iran. *BMC Microbiology*.

[45] Shabayek S., Ferrieri P., Spellerberg B. (2021). Group B streptococcal colonization in african countries: prevalence, capsular serotypes, and molecular sequence types. *Pathogens*.

[46] Doran K. S., Liu G. Y., Nizet V. (2003). Group B streptococcal beta-hemolysin/cytolysin activates neutrophil signaling pathways in brain endothelium and contributes to development of meningitis. *Journal of Clinical Investigation*.

[47] Harris T. O., Shelver D. W., Bohnsack J. F., Rubens C. E. (2003). A novel streptococcal surface protease promotes virulence, resistance to opsonophagocytosis, andcleavage of human fibrinogen. *Journal of Clinical Investigation*.

[48] Nizet V., Gibson R. L., Chi E. Y., Framson P. E., Hulse M., Rubens C. E. (1996). Group B streptococcal beta-hemolysin expression is associated with injury of lung epithelial cells. *Infection and Immunity*.

[49] Wang Z., Guo C., Xu Y., Liu G., Lu C., Liu Y. (2014). Two novel functions of hyaluronidase from Streptococcus agalactiae are enhanced intracellular survival and inhibition of proinflammatory cytokine expression. *Infection and Immunity*.

[50] Lin F. P.-Y., Lan R., Sintchenko V., Gilbert G. L., Kong F., Coiera E. (2011). Computational bacterial genome-wide analysis of phylogenetic profiles reveals potential virulence genes of Streptococcus agalactiae of Streptococcus agalactiae. *PLoS One*.

[51] Zhang Z. (2021). Research advances on Tilapia streptococcosis. *Pathogens*.

[52] Hossain A., Habibullah-Al-Mamun M., Nagano I., Masunaga S., Kitazawa D., Matsuda H. (2022). Antibiotics, antibiotic-resistant bacteria, and resistance genes in aquaculture: risks, current concern, and future thinking. *Environmental Science and Pollution Research*.

[53] Rico A., Oliveira R., McDonough S. (2014). Use, fate and ecological risks of antibiotics applied in tilapia cage farming in Thailand. *Environmental Pollution*.

[54] Chen J., Sun R., Pan C., Sun Y., Mai B., Li Q. X. (2020). Antibiotics and food safety in aquaculture. *Journal of Agricultural and Food Chemistry*.

[55] Pepi M., Focardi S. (2021). Antibiotic-resistant bacteria in aquaculture and climate change: a challenge for health in the mediterranean area. *International Journal of Environmental Research and Public Health*.

[56] Fry J. P., Cabello F., Good C. M., Lunestad B. T. (2020). Veterinary drug use in United States net pen Salmon aquaculture: implications for drug use policy, Love. *Aquaculture*.

[57] Manage P. M. (2018). Heavy use of antibiotics in aquaculture: emerging human and animal health problems – a review. *Sri Lanka Journal of Aquatic Sciences*.

[58] Heuer O. E., Kruse H., Grave K., Collignon P., Karunasagar I., Angulo F. J. (2009). Human health consequences of use of antimicrobial agents in aquaculture. *Clinical Infectious Diseases*.

[59] Miranda C. D., Godoy F. A., Lee M. R. (2018). Current status of the use of antibiotics and the antimicrobial resistance in the Chilean salmon farms. *Frontiers in Microbiology*.

[60] Ikeogu C., Fc F., Nsofor C., Ikpeze O. A review of risk factors for fish diseases in aquatic environments.

[61] Phuoc N. N., Linh N. T. H., Crestani C., Zadoks R. N. (2021). Effect of strain and enviromental conditions on the virulence of Streptococcus agalactiae (Group B Streptococcus; GBS) in red tilapia (Oreochromis sp.). *Aquaculture*.

[62] Kayansamruaj P., Pirarat N., Hirono I., Rodkhum C. (2014). Increasing of temperature induces pathogenicity of Streptococcus agalactiae and the up-regulation of inflammatory related genes in infected Nile tilapia (Oreochromis niloticus). *Veterinary Microbiology*.

[63] Chideroli R. T., Amoroso N., Mainardi R. M. (2017). Emergence of a new multidrug-resistant and highly virulent serotype of Streptococcus agalactiae in fish farms from Brazil. *Aquaculture*.

[64] Chen M., Wang R., Li L.-P. (2012). Screening vaccine candidate strains against Streptococcus agalactiae of tilapia based on PFGE genotype. *Vaccine*.

