# Supplementary material for: Streptococcus agalactiae Serotype VII, an Emerging Pathogen Affecting Snakeskin Gourami (Trichogaster pectoralis) in Intensive Farming
Source: Transbound Emerg Dis. 2023 Mar 24;2023:1682047. doi: 10.1155/2023/1682047 (PMC12016886; doi:10.1155/2023/1682047)
Supplement: Supplementary Materials — The supplementary file contains three figures and four tables. Figure S1. A map of Thailand showing the locations of sample collection. Figure S2. Identification of bacterial samples using a multiplex PCR assay with specific primers against S. agalactiae, S. iniae, and Lactococcus garvieae. Figure S3. Molecular serotyping of S. agalactiae using a multiplex PCR assay with specific primers based on capsular polysaccharide (cps) cluster gene. Table S1. Detail of locations, farming history, mortality, and other pathogens detected that were observed in moribund snakeskin gourami in this study. Table S2. List of primers used in this study. Table S3. Diameter size and interpretation of antimicrobial susceptibility test (zone of diameter breakpoint) and the zone diameter of the standard S. pneumoniae strain ATCC® 49619. Table S4. The median lethal dose (LD50) dose for three representative isolates of S. agalactiae (KU63SA1, KU63SA2, and KU64SA10). [file 1682047.f1.docx]

**Supplementary Materials (Figures and Tables)**


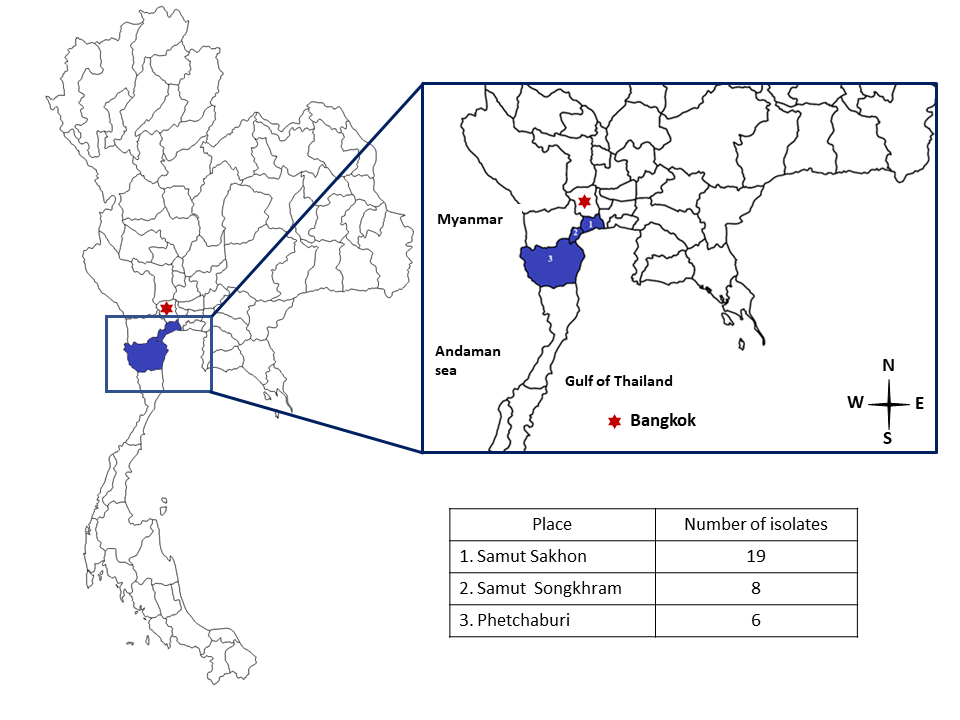


**Figure S1** A map of Thailand showing the locations of sample collection. Three provinces (Samut Sakhon, Samut Songkhram, and Phetchaburi provinces) are highlighted.


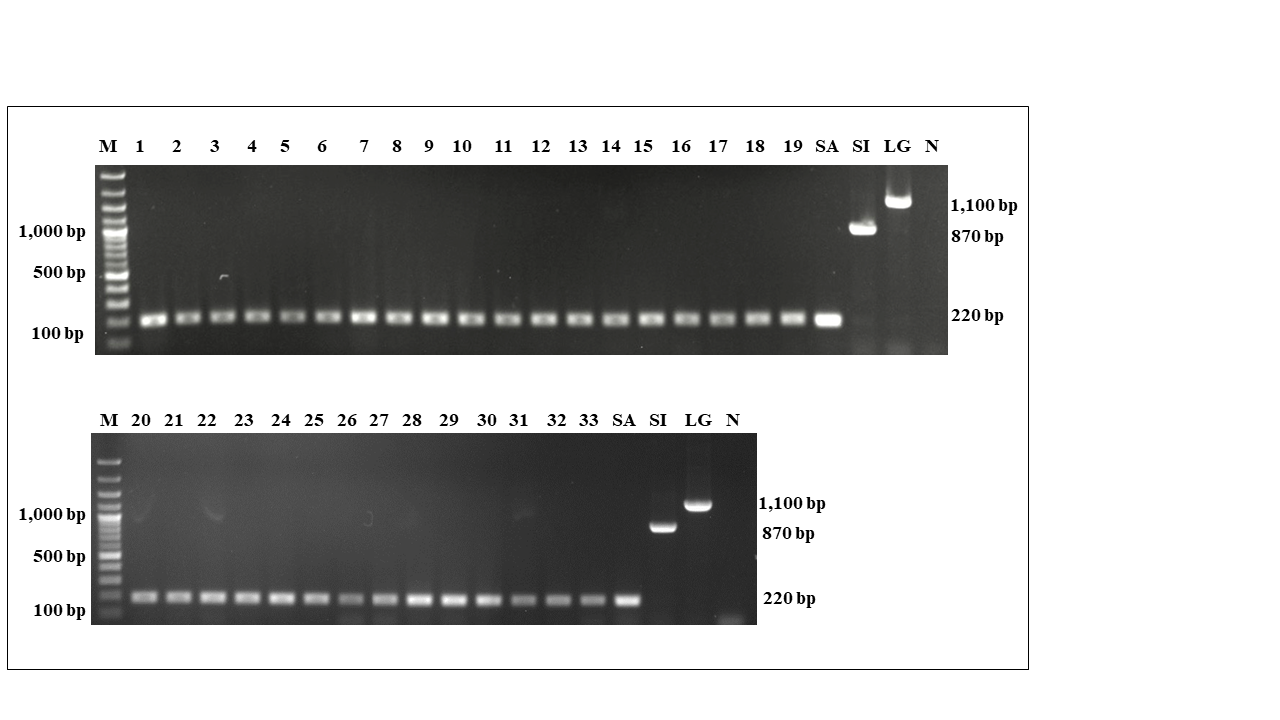


**Figure S2** Identification of bacterial samples using a multiplex PCR assay with specific primers against *S. agalactiae*, *S. iniae* and *L. garvieae*. The positive controls (*S. agalactiae*, *S. iniae* and *L. garvieae*) were previously characterized and provided by the Kamphaeng Saen Veterinary Diagnostic Center, Faculty of Veterinary Medicine, Kasetsart University. Lane M = 100 bp Plus DNA ladder, Lane 1–4 = KU63SA1–KU63SA4, Lane 5–22 = KU64SA1– KU64SA18, Lane 23–33 = KU65SA1–KU65SA11, SA = a positive control of *S. agalactiae*, SI = a positive control of *S. iniae*, LG = a positive control of *L. garvieae*, N = negative control


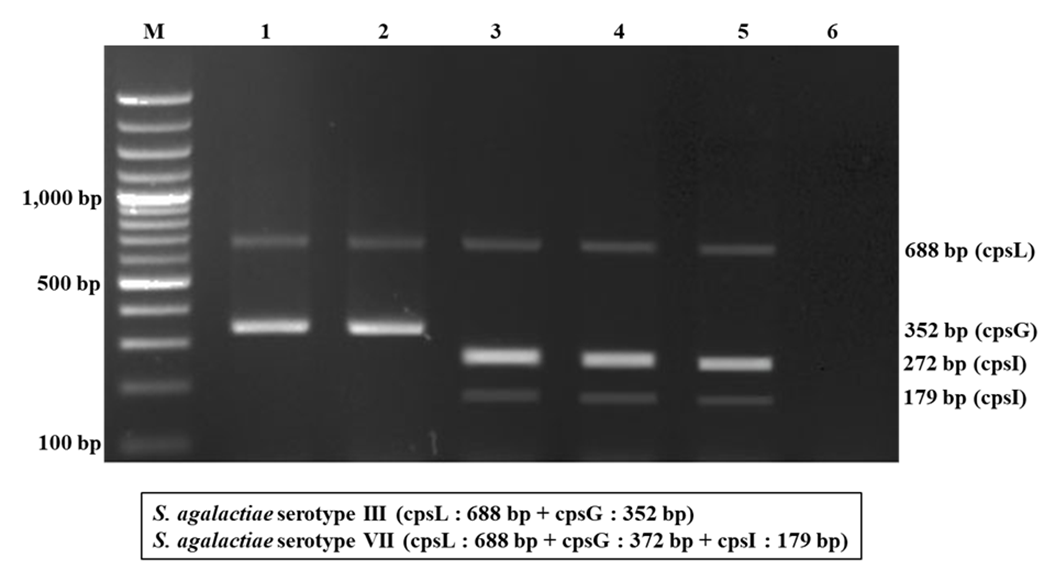


**Figure S3** Molecular serotyping of representative isolates of *S. agalactiae* using a multiplex PCR assay with specific primers based on capsular polysaccharide (*cps*) cluster gene. The serotype of representative *S. agalactiae* was determined based on the size of the PCR products. Lane M = 100 bp Plus DNA ladder, Lane 1 and 2 = *S. agalactiae* seroype III previously isolated from Nile tilapia and red tilapia (size of PCR products: cpsL (688 bp), cpsG (352 bp), Lane 3–5 = representative isolates of *S. agalactiae* seroype VII isolated from snakeskin gourmai KU63SA1 (Samut Sakhon), KU63SA2 (Samut Songkhram), and KU64SA10 (Phetchaburi) (size of PCR products: cpsL (688 bp), cpsG (252 bp), and cps I (179 bp), Lane 6 = negative control.

**Table S1** Detail of locations, farming history, mortality and other pathogens detected that were observed in moribund snakeskin gourami in this study.

| Sample code | Year | Farm location | Farm | Size of pond  (Arce) | Age (month) | Stock density  (Fish/Acre) | Mortality  (%) | Parasite | Bacteria |
| --- | --- | --- | --- | --- | --- | --- | --- | --- | --- |
| KU63SA1 | 2020 | Samut Sakhon | 1 | 7.2 | 4.5 | 25,000 | 28 | - | *S. agalactaie* |
| KU63SA2 | 2020 | Samut Songkhram | 2 | 12 | 7 | 25,000 | 45 | - | *S. agalactiae* |
| KU63SA3 | 2020 | Samut Sakhon | 3 | 8 | 5 | 25,000 | 23 | *Trichodina* spp. | *S. agalactiae* |
| KU63SA4 | 2020 | Samut Sakhon | 4 | 8 | 7 | 20,000 | 20 | *Trichodina* spp. | *S. agalactiae* |
| KU64SA1 | 2021 | Samut Sakhon | 5 | 3.2 | 4.5 | 56,250 | 37 | *Gyrodactylus* spp.  *Trichodina* spp. | *S. agalactiae*  *Aeromonas* spp. |
| KU64SA2 | 2021 | Samut Sakhon | 5 | 3.2 | 4.5 | 56,250 | 23.5 | *Trichodina* spp. | *S. agalactiae*  *Aeromonas* spp. |
| KU64SA3 | 2021 | Samut Songkhram | 6 | 8 | 5 | 25,000 | 20 | - | *S. agalactiae* |
| KU64SA4 | 2021 | Samut Songkram | 7 | 10 | 2 | 32,000 | 23 | *Trichodina* spp. | *S. agalactiae*  *Aeromonas* spp. |
| KU64SA5 | 2021 | Samut Songkhram | 7 | 15 | 2 | 32,000 | 20 | - | *S. agalactiae*  *Aeromonas* spp. |
| KU64SA6 | 2021 | Samut Songkhram | 8 | 10.8 | 1 | 27,700 | 35 | *Trichodina* spp. | *S. agalactiae*  *Flavobacterium columnare* |
| KU64SA7 | 2021 | Samut Songkhram | 9 | 10.8 | 1 | 93,750 | 30 | - | *S. agalactiae* |
| KU64SA8 | 2021 | Samut Sakhon | 10 | 2 | 4 | 25,000 | 30 | - | *S. agalactiae* |
| KU64SA9 | 2021 | Samut Sakhon | 10 | 1.6 | 3 | 27,700 | 28 | - | *S. agalactiae* |
| KU64SA10 | 2021 | Phetchaburi | 11 | 8.8 | 3 | 50,000 | 34 | - | *S. agalactiae*  *Aeromonas* spp*.* |
| KU64SA11 | 2021 | Phetchaburi | 11 | 8 | 3 | 50,000 | 27 | - | *S. agalactiae* |
| KU64SA12 | 2021 | Samut Sakhon | 12 | 4 | 5 | 37,500 | 24 |  | *S. agalactiae*  *Aeromonas* spp. |
| KU64SA13 | 2021 | Samut Sakhon | 12 | 10 | 8 | 35,000 | 35 | Henneguya spp. | *S. agalactiae* |
| KU64SA14 | 2021 | Samut Sakhon | 13 | 7.2 | 4 | 75,000 | 28 | - | *S. agalactiae* |
| KU64SA15 | 2021 | Phetchaburi | 14 | 4.8 | 5 | 58,333 | 20 | - | *S. agalactiae*  *Aeromonas* spp. |
| KU64SA16 | 2021 | Phetchaburi | 14 | 8 | 5 | 43,750 | 27 | - | *S. agalactiae* |
| KU64SA17 | 2021 | Samut Sakhon | 15 | 4 | 4 | 50,000 | 22 | - | *S. agalactiae* |
| KU64SA18 | 2021 | Samut Sakhon | 15 | 4 | 5.5 | 50,000 | 40 | *Trichodina* spp. | *S. agalactiae*  *Aeromonas* spp. |
| KU65SA1 | 2022 | Samut Sakhon | 16 | 2 | 7 | 40,000 | 32 | *-* | *S. agalactiae*  *Aeromonas* spp. |
| KU65SA2 | 2022 | Samut Sakhon | 16 | 2.8 | 5 | 45,000 | 45 | *Trichodina* spp*. Henneguya* spp. | *S. agalactiae*  *Aeromonas* spp. |
| KU65SA3 | 2022 | Samut Songkhram | 17 | 6.4 | 4 | 22,500 | 23 | *-* | *S. agalactiae* |
| KU65SA4 | 2022 | Phetchaburi | 18 | 4 | 7 | 45,000 | 28.6 | *-* | *S. agalactiae* |
| KU65SA5 | 2022 | Phetchaburi | 18 | 5 | 6 | 47,000 | 25 | *-* | *S. agalactiae Aeromonas* spp*.* |
| KU65SA6 | 2022 | Samut Sakhon | 19 | 10.8 | 4 | 44,200 | 20 | *Henneguya* spp. | *S. agalactiae*  *Aeromonas* spp. |
| KU65SA7 | 2022 | Samut Sakhon | 19 | 2.8 | 6 | 30,000 | 38 | *-* | *S. agalactiae*  *Aeromonas* spp. |
| KU65SA8 | 2022 | Samut Sakhon | 20 | 3.6 | 5 | 33,333 | 35 | *Trichodina* spp. | *S. agalactiae*  *Aeromonas* spp*.* |
| KU65SA9 | 2022 | Samut Sakhon | 20 | 2.8 | 5 | 33,333 | 30 |  | *S. agalactiae*  *Aeromonas* spp |
| KU65SA10 | 2022 | Samut Sakhon | 21 | 3.2 | 7 | 62,500 | 36 |  | *S. agalactiae* |
| KU65SA11 | 2022 | Samut Songkhram | 22 | 12 | 5 | 35,000 | 37.3 |  | *S. agalactiae* |

**Table S2** List of primers used in this study.

| Gene | Primer name | Sequence (5’ to 3’) | References |
| --- | --- | --- | --- |
| 16S rRNA  (*S. agalactiae*) | F1 | GAGTTTGATCATGGCTCAG | [25] |
|  | IMOD | ACCAACATGTGTTAATTACTC |  |
| *lct*O  (*S. iniae*) | LOX-1 | AAGGGGAAATCGCAAGTGCC | [26] |
|  | LOX-2 | ATATCTGATTGGGCCGTCTAA |  |
| 16S rDNA  (*L. garvieae*) | pLG-1 | CATAACAATGAGAATCGC | [27] |
|  | pLG-2 | GCACCCTCGCGGGTTG |  |
| 16S rRNA | EubB | AGAGTTTGATCMTGGCTCAG | [29] |
|  | EubA | AAGGAGGTGATCCANCCRCA |  |
| Capsular | *cpsI*-Ia-6-7-F | GAATTGATAACTTTTGTGGATTGCGATGA | [18] |
| polysaccharide | *cpsI*-6-R | CAATTCTGTCGGACTATCCTGATG |  |
| (*cps*) | *cpsI*-7-R | TGTCGCTTCCACACTGAGTGTTGA |  |
|  | *cpsL*-F | CAATCCTAAGTATTTTCGGTTCATT |  |
|  | *cpsL*-R | TAGGAACATGTTCATTAACATAGC |  |
|  | *cpsG*-F | ACATGAACAGCAGTTCAACCGT |  |
|  | *cpsG*-R | ATGCTCTCCAAACTGTTCTTGT |  |
|  | *cpsG*-2-3-6-R | TCCATCTACATCTTCAATCCAAGC |  |
|  | *cpsN*-5-F | ATGCAACCAAGTGATTATCATGTA |  |
|  | *cpsN*-5-R | CTCTTCACTCTTTAGTGTAGGTAT |  |
|  | *cpsJ*-8-F | TATTTGGGAGGTAATCAAGAGACA |  |
|  | *cpsJ*-8-R | GTTTGGAGCATTCAAGATAACTCT |  |
|  | *cpsJ*-2-4-F | CATTTATTGATTCAGACGATTACATTGA |  |
|  | *cpsJ*-2-R | CCTCTTTCTCTAAAATATTCCAACC |  |
|  | *cpsJ*-4-R | CCTCAGGATATTTACGAATTCTGTA |  |
|  | *cpsI*-7-9-F | CTGTAATTGGAGGAATGTGGATCG |  |
|  | *cpsI*-9-R | AATCATCTTCATAATTTATCTCCCATT |  |
|  | *cpsJ*-Ib-F | GCAATTCTTAACAGAATATTCAGTTG |  |
|  | *cpsJ*-Ib-R | GCGTTTCTTTATCACATACTCTTG |  |
| *cyl*E | *cyl*E-F | GTACATTAGGTGCCTTTGG | [19] |
|  | *cyl*E-R | TACTCAGCCTTTCTCCATC |  |
| *hyl*B | *hyl*B-F | TCTATGCTGACGGTTCTTAC | [19] |
|  | *hyl*B-R | GAGGTCTAAGTTTCGCTCTT |  |
| *scp*B | s*cp*B-F | ACAACGGAAGGCGCTACTGTTC | [30] |
|  | *scp*B-R | ACCTGGTGTTTGACCTGAACTA |  |
| *csp*A | *csp*A-F | CTGCTAAAGCACACCTAAAC | [19] |
|  | *csp*A-R | ATCAGTAGTGGTTCCTTTCC |  |
| *cfb* | *cfb*-F | AAGCGTGTATTCCAGATTTCCT | [22] |
|  | *cfb-*R | CAGTAATCAAGCCCAGCAA |  |

**Table S3** Diameter size and interpretation of antimicrobial susceptibility test (zone of diameter breakpoint) and the zone diameter of the standard *S. pneumoniae* strain ATCC^®^ 49619

| Antimicrobial agents | Disc | Zone of diameter breakpoint (mm)^a^ | | | Diameter zone of  *S. pneumoniae strain* ATCC^®^ 49619 (mm) | Interpretation |
| --- | --- | --- | --- | --- | --- | --- |
|  | content (μg) | R | I | S |  |  |
| Amoxicillin | 10 | -^b^ | -^b^ | ≥24 | 30 | S |
| Erythromycin | 15 | ≤15 | 16–20 | ≥21 | 24 | S |
| Enrofloxacin | 5 | ≤12 | 13–15 | ≥16 | 20 | S |
| Doxycycline | 30 | ≤18 | 19–22 | ≥23 | 29 | S |
| Oxytetracycline | 30 | ≤18 | 19–22 | ≥23 | 33 | S |
| Sulfamethoxazole- trimethoprim | 25 | ≤15 | 16–18 | ≥19 | 25 | S |

Abbreviation:

^a^Zone diameter breakpoints for *S. agalactiae* obtained from snakeskin gourami were determined according to the standards established for the *Streptococcus* spp. β-hemolysis group (amoxicillin, erythromycin, enrofloxacin, doxycycline and oxytetracycline) and for *Streptococcus pneumoniae* (sulfamethoxazole -trimethoprim) as specified in the CLSI guideline [33].

^b^not defined.

The categorical interpretations: R = resistant, I = immediate susceptible, S = susceptible

**Table S4** The median lethal dose (LD_50_) dose for three representative isolates of *S. agalactiae* (KU63SA1, KU63SA2, and KU64SA10).

| Bacterial isolates | Source | LD_50_ (CFU/fish) |
| --- | --- | --- |
| KU63SA1 | Samut Sakhon | 8.09 × 10^6^ |
| KU63SA2 | Samut Songkhram | 8.23 × 10^6^ |
| KU64SA10 | Phetchaburi | 8.17 × 10^6^ |
